# Emotionality Modulates the Effect of Chronic Stress on Feeding Behaviour in Birds

**DOI:** 10.1371/journal.pone.0087249

**Published:** 2014-02-03

**Authors:** Angélique Favreau-Peigné, Ludovic Calandreau, Paul Constantin, Bernard Gaultier, Aline Bertin, Cécile Arnould, Agathe Laurence, Marie-Annick Richard-Yris, Cécilia Houdelier, Sophie Lumineau, Alain Boissy, Christine Leterrier

**Affiliations:** 1 UMR 791 Modélisation Systémique Appliquée aux Ruminants, AgroParisTech, Paris, France; 2 UMR 791 Modélisation Systémique Appliquée aux Ruminants, INRA, Paris, France; 3 UMR 85 Physiologie de la Reproduction et des Comportements, INRA, Nouzilly, France; 4 UMR 6175, CNRS, Nouzilly, France; 5 Université de Tours, Tours, France; 6 IFCE, Nouzilly, France; 7 UE 1295 Pôle d’Expérimentation Avicole de Tours, INRA, Nouzilly, France; 8 Ethos UMR 6552, Université de Rennes 1, CNRS, Rennes, France; 9 UMR 1213 Herbivores, INRA, St-Genès Champanelle, France; Pennsylvania State University, United States of America

## Abstract

Chronic stress is a long-lasting negative emotional state that induces negative consequences on animals’ psycho-physiological state. This study aimed at assessing whether unpredictable and repeated negative stimuli (URNS) influence feeding behaviour in quail. Sixty-four quail were exposed to URNS from day 17 to 40, while 64 quail were undisturbed. Two lines divergently selected on their inherent emotionality were used to assess the effect of genetic factors on the sensitivity to URNS. All quail were submitted to a sequential feeding procedure (using two diets of different energetic values) which placed them in a contrasting situation. Behavioural tests were performed to assess the emotional reactivity of the two lines. Results confirmed that differences exist between them and that their emotional reactivity was enhanced by URNS. Diet preferences, motivation and daily intake were also measured. URNS did not change the preferences for the hypercaloric diet compared to the hypocaloric diet in choice tests, but they reduced daily intakes in both lines. Motivations for each diet were differently affected by URNS: they decreased the motivation to eat the hypercaloric diet in quail selected for their low inherent fearfulness whereas they increased the motivation to eat the hypocaloric diet in quail selected for their high inherent fearfulness, which suggested a devaluation process in the former and a compensatory behaviour in the later. Growth was furthermore reduced and laying delayed by URNS in both lines. In conclusion, the exposure to URNS induced interesting changes in feeding behaviour added with an increase in emotional reactivity and an alteration of production parameters. This confirms that both lines of quail experienced a chronic stress state. However differences in feed motivation and emotional reactivity between lines under chronic stress suggested that they experienced different emotional state and use different ways to cope with it depending on their genetic background.

## Introduction

Chronic stress is a long-lasting negative emotional state. It is induced by animal’s exposure to unpredictable and repeated negative stimuli (URNS) in various species such as rodents [1], [2], livestock mammals [3], [4] and birds [5], [6]. Chronic stress induces numerous and various negative consequences on animals’ psycho-physiological state. For instance, it decreases reproductive success [7] and body weight in starlings [8], delays the onset of lay and decreases egg production in hens [9], and affects immune system in Japanese quail [10]. Chronic stress can also damage animal behaviour such as emotionality [11] and feeding behaviour. On that last point, chronic stress could have various, even opposite, consequences: animals can indeed show an increase or a decrease in their daily intake [12], and/or show anhedonia (*i.e.* the inability to experience pleasure; [13]), and/or search for a comfort food (*i.e.* a simple, palatable, and high-energy food; [14]). The nature of the stressor (physical or emotional stressor) is responsible for the different responses observed while testing for anhedonia in rats [15]. Recent studies have also suggested that individual factors such as personality and/or genetic factors are able to modulate the occurrence of depression and the resilience to stress in humans [16]. This influence of genetic factors on the susceptibility to chronic stress is well established in rodents [17], [18], including the susceptibility to express anhedonia as a consequence of chronic mild stress [19].

Various strains of rodents were selected and helped to demonstrate the link between their inherent fearfulness and their vulnerability to chronic stress. For instance, the BALB/cByJ mice exhibited a high level of anxiety in several behavioural tests (the elevated plus maze, the open-field test, …) and expressed marked behavioural and neurochemical changes in response to a chronic stress procedure [20]. In birds, an original model is available as a divergent selection for duration of tonic immobility has resulted in two divergent genetic lines: the LTI and the STI lines. They display long and short duration of tonic immobility respectively, but different behaviours in many other stressful situations. LTI quail were less active when restrained in a crush cage [21] and showed a higher fear-behaviour frequency after the approach of a man [22] compared to STI quail. Concerning their responses to novelty, LTI quail showed a higher reluctance to enter in a novel environment as well as a higher behavioural inhibition in the open field [23], [24], and they were more frightened by a novel food [25] and by a novel object placed in their home cage [26] compared to STI quail. Altogether, these findings have led to the assumption that the selection for tonic immobility generally affects the emotionality of birds (*i.e.* the propensity to be more or less easily frightened).

With various chronic stress procedures lasting from 8 to 15 days, some studies demonstrated that quail’s exposed to unpredictable and repeated negative stimuli (URNS) rested more [27], showed lower immune responses [10] and lower body weight in some cases [28], and showed altered spatial learning and spatial reversal learning tasks [27], [28]. They also demonstrated that quail are differently affected by URNS exposures depending on genetic factors: quail selected for their high inherent fearfulness (LTI) had lower plasma corticosterone levels [29] and higher emotionality in open field tests [27], [29] after exposure to URNS while they were no effect of URNS exposures in quail selected for their low inherent fearfulness.

Both the differences in inherent emotionality of the two lines after exposure to URNS and the induction of a chronic stress by the exposure to URNS were assessed throughout common production parameters (growth, egg production) and behavioural reactivity tests. Then, this study aimed at assessing whether URNS can alter feeding behaviour in quail and whether it can be modulated by their different inherent emotionality. Because a high variability is described in humans and rodents concerning the anhedonic response to chronic mild stress [30], we chose to use two diets (a highly palatable one and a low palatable one) and several measures concerning the feeding behaviour of the birds. We thus measured food preferences in a two-way choice test which allows a simultaneous comparison of the two diets, short-term intakes of each diet to assess quail’s motivations to eat each one, and daily intakes of each diet since it involves metabolic regulations.

## Materials and Methods

The study was conducted indoors at the Pôle d’Expérimentation Avicole de Tours (UE PEAT, INRA, Nouzilly, France) between September and November 2010.

### Ethics Statement

The experimental procedure has been submitted to and validated by the Ethical Regional Committee (ref. 2010–23).

### Animals and Housing

Female Japanese quail (*Coturnix japonica*) selected for their long (n = 64) or short (n = 64) duration of tonic immobility (LTI and STI respectively) were used during this experiment. Tonic immobility duration is a standard and robust measure of bird fearfulness (see [31] for a full description of the lines). From the day of hatching (day 0) to day 15, chicks were reared in two communal pens depending on their genetic line but they were all placed in the same room so that to ensure similar environmental conditions. They were first maintained under a temperature of 40°C and under continuous illumination, and then the temperature and the photoperiod were adjusted progressively to 24°C and a 12∶12 h light/dark schedule (light on at 8∶30 am). Chicks were wing-banded at 2 days of age. At day 15, chicks were sexed thanks to their feather dimorphism and weighed before being transferred in single home cages (35×25×21 cm) placed in two different rooms depending on their experimental groups. Temperature and light conditions were similar in both groups (*i.e.* 25±2°C and a 12∶12 h light/dark schedule). Water was available *ad libitum*.

Each individual cage contained a plastic square mesh on the whole surface of the ground and a rectangle of artificial turf on half the whole surface of the ground to provide a pleasant contact to sit on. Opaque plastic covered half the surface of the front lateral sides of the cage and was also put at the front exterior of the cage so that quail could not observe her neighbour eating whereas she can see her at the back side of the cage.

### Feeding Procedure and Measurements

During all the experiment, food was offered *ad libitum* to the animals at 9∶30 am (*i.e.* one hour after the start of the light phase).

A standard food for quail chicks was first used until Day 7 (Stargib G111®, Evialis, France). Then, three diets were used in this experiment: a normocaloric diet which corresponded to the diet commonly used in quail (Metabolizable energy, ME = 12.56 MJ), a hypercaloric diet (ME = 13.39 MJ) and a hypocaloric diet (ME = 11.72 MJ). As mentioned into brackets, they differed in their energetic content whereas they were iso-proteic (Crude Protein = 190 g/kg) and contained the same amount of lysine (10.7 g/kg) which is known to largely influence feeding behaviour in birds [32], [33]. The composition of the diets was established according to Bouvarel et al [34]; it aimed at providing a highly palatable diet to the birds (the hypercaloric diet) and an energetic contrast between the hyper- and hypocaloric diets. The hypercaloric diet was considered as a comfort food (*i.e.* a simple, palatable, and high-energy food; [14]). The feeding procedure and measurements are summed up in Table 1.

**Table 1:** Feeding procedure and measures depending on the age of the quail. URNS were applied from day 17 to day 44.

Days of age		Food^a^	Measures
0 to 7		Growth	
8		Hypo	
9		Hyper	
10		Hypo	
11		Hyper	
12		Hypo	
13		Hyper	
14 to 16		Normo	TI (d15)^b^, Body weight (d15), Transfer in individual cages (d16)
Period 1	Period 2		
17	28	Hypo	Food intake (30 min, 24 h)
18	29	Hyper	Food intake (30 min, 24 h)
19	30	Hypo	Food intake (30 min, 24 h)
20	31	Hyper	Food intake (30 min, 24 h)
21	32	Hypo	Food intake (30 min, 24 h)
22	33	Hyper	Food intake (30 min, 24 h)
23	34	Hypo	Food intake (30 min, 24 h)
24	35	Hyper	Food intake (30 min, 24 h)
25	36	Normo	Food intake (30 min, 24 h)
26	37	Normo	Food intake (30 min, 24 h)
27	38	Choice test - Normo	Food preference, Body weight
39–40		Normo	
41		Normo	Food neophobia
42		Normo	Constraint in a crush cage
43		Normo	Reactivity to human
44		Normo	Open field
45		Normo	Reactivity to novel object
46 to the end		Normo	Body weight (d49+d59), Laying, Food preference (d55)

a Different types of food were used: a grower food, a normocaloric, a hypocaloric and a hypercaloric diets. When not mentioned, the last three diets were used in pelleted form.

b TI: Tonic Immobility test.

From the day of hatching to day 15, before the exposure to URNS, chicks were fed in group. From day 8 to day 13, they were fed the hypocaloric diet on even days and the hypercaloric diet on odd days. It was a simple food learning task: each day, quail ate one diet and experienced its associated post-ingestive consequences so that they can learn the post-ingestive value of each diet. This sequential feeding procedure is known to induce preferences for the hypercaloric diet in broilers [34]. At day 14, 15 and 16, they were fed a normocaloric diet.

From day 16 to the end, animals lived in individual cages and were thus fed individually.

From day 17 to day 24 (period 1), animals were offered 20 g of the hypocaloric diet on odd days and 20 g of the hypercaloric diet on even days. This feeding schedule was repeated once during period 2, from day 28 to day 35. Each day, the same procedure was established. One diet was offered from 9∶30 am to 10∶00 am, then each feeder was removed, the potential food wastage was collected and they were weighed altogether to measure short-term intake which is an indirect measure of the motivation to eat each diet [35]. At 10∶30 am, each feeder was offered again and was removed on the next day, just before light turned on, and weighed with the potential food wastage in order to measure daily intake. Because of the daily basis of this measurement, it took into account the physiological regulations; it was more what the animal needed than what it liked. One hour of fasting came before diet offer so that to decrease individual variability in the motivation to eat. An individual mean daily intake of each diet was calculated over each period to perform statistical analysis. Similarly an individual mean short-term intake was calculated over each period to measure motivation for each diet.

At the end of each period, quail received the normocaloric diet for 3 days in order to test diet preferences in animals with balanced energy and metabolic states. In the morning of the 3^rd^ day with the normocaloric diet (day 27 and 38), quail were submitted to a choice test between the hypocaloric and hypercaloric diets, offered simultaneously from 9∶30 am to 10∶00 am. After 30 minutes, the feeders were removed and were weighed to measure the intake of each diet. As the food wastages of the two diets were intermixed, they were not use in the measure. The relative preference of each diet was assessed as a proportion of total food intake. At 10∶30 am, the normocaloric diet was offered until next day morning, just before the light turned on. Because the normocaloric diet was used to induce an energetic and metabolic stable state before testing diet preferences between the hypocaloric and the hypercaloric diets, and because daily normocaloric diet intakes were influenced by the various consumptions during these tests, they were not compared between groups.

From day 39 to the end of the experiment, quail were offered the normocaloric diet. An additional choice test was performed at day 55.

The normocaloric diet was offered in a green feeder placed in a central position, in front of the cage. For half the STI and LTI quail, a blue feeder was associated with the hypocaloric diet and a white feeder was associated with the hypercaloric diet; and the reverse for the other half. For a given animal, the blue feeder and the white feeder were always placed in the same position (right or left), in front of the cage. These colours and positions cues aimed at helping the animal discriminating and learning between the different food items. The colour (blue or white) and the position (right or left) of the feeder as well as the nature of the diet associated with it were balanced between groups and genetic lines. A kind of plastic gutter was placed below the feeder to collect food in case of wastage.

### Chronic Stress Procedure

The chronic stress procedure was adapted from studies in starlings [6], [7], sheep [3] and Japanese quail [27], [29].

At 15^th^ day of age, quail were divided in two groups, *i.e.* control and disturbed groups (32 LTI and 32 STI quail in each group), balanced on their body weight and tonic immobility response. Female quail of both lines were either confronted with unpredictable and repeated negative stimuli (disturbed group) or left undisturbed (control group) from the age of 17 to 44 days. From the age of 17 to 40 days, quail from the disturbed group were exposed to URNS four times per day and once per night while quail from the control group were just visited by a human four times per day. For the last four days of the chronic stress procedure, the number of negative stimuli per day was reduced and adapted to the length of the behavioural tests. Each negative stimulus lasted 30 minutes, continuously or not. They were of seven different types: 1/confinement in a corner of the home cage, 2/disturbances in the home cage (*e.g.* cage tapping, waving of a plastic flag above the cages, air and water spraying on the animal, plastic stick banged on the rod of the cage or put into the cage without touching the animal; they were performed at random during the sessions), 3/cage shaking, 4/noises (*i.e.* different noises - human voice, alert call from a quail, doorbell, barking… - of different durations broadcasted at random during the 30 min sessions), 5/crowding (*i.e.* quail were put together in a plastic poultry cage without mixing the lines), 6/novel environment (placed individually in buckets in a novel room), and 7/transport while put together in a plastic cage. The experimenters wore a green lab coat to apply URNS on disturbed quail whereas they wore a yellow lab coat in all other cases (*e.g.* measurements, care, etc.).

URNS and visits occurred at random times, and a given URNS was never used twice per day in order to increase unpredictability and decrease animal habituation to the stress procedure. Moreover, URNS were delivered manually or automatically to decrease the possible association between human presence and the occurrence of URNS. During the light phase, both manually and automatically generated URNS were delivered whereas only automatically generated stressors (noises and cage shaking) were used during the dark phase.

Control birds were visited while URNS were applied to disturbed birds, and their feeders were removed to avoid feeding while disturbed quail were not allowed to feed (during confinement in a corner, crowding in a plastic poultry cage, transport).

### Behavioural Measures

From day 41 to day 45, behavioural tests assessing emotional reactivity were performed daily (Table 1).

#### A. Reactivity to a novel food

Food neophobia was tested at 41 days of age. The new food consisted in crushed corn. As it is a new food, it is assumed that its post-ingestive value is unknown by the naïve animal. Their familiar food (*i.e.* the normocaloric diet) was removed just before the light turned on. One hour after the light turned on, the new food was offered during 30 minutes in their familiar green feeder situated in a central position, in front of the cage. At the end of the test, the feeders were removed, the potential food wastage was collected and they were weighed altogether to measure the amount of crushed corn eaten by the quail.

#### B. Restraint in a crush-cage

At 42 days of age, quail were individually placed in a box and then carried to a novel room containing no other bird. There, they were transferred during 10 minutes in a crush-cage consisting in a wooden box (11×11×11 cm) closed by a wire mesh at the top of it. Animal activity (body movement or totally motionless, *i.e.* no movement of the animal’s head and body) was recorded thanks to a camera place above the crush-cage, and a scan sampling was performed with an interval of 30 seconds between two observations.

#### C. Reactivity to human

Reactivity to human was tested at 43 days of age. Two experimenters tested simultaneously the two groups of quail. Experimenter came in front of a first column of 8 quail equipped with 4 camera fixed at different vertical levels on a metal bar. Once placed in front of the cage, the experimenter stayed motionless during 2 minutes. Quail were filmed during this interval. Then, the experimenters went outside of the rooms and entered in the alternate room to test 8 other quail. The procedure was repeated 8 times to test all the quail.

A continuous sampling method was then used to note whether the animal was far or close to the human. Each time the quail cross a line which artificially divided the cage in two parts (front and back) with both feet, it was considered changing its front/back location. The duration spent in each part of the cage and the latency to come in front of the cage were then measured.

#### D. Reactivity to a novel environment

The open field test was performed at 44 days of age. It allowed assessing reactivity to a novel environment, *i.e.* an arena (80 × 80 × 50 cm) made of wood, with a beige lino on the floor. This arena was surrounded by a green curtain to avoid the quail escaping from it during the test. Three similar arena were placed in three different rooms, with similar temperature and light conditions, so that three quail were tested simultaneously.

Animals were individually placed in a box and carried to a novel room containing no other bird. Each quail was then placed at the center of the open field arena and left alone for 6 minutes. A digital camera was fixed above the arena to record the location of the birds. Images were captured at a rate of 5 Hz and transmitted to a computer running the Ethovision tracking system (v. XT 7 Noldus Technology, Wageningen, The Netherland).

#### E. Reactivity to a novel object

All quail were tested in their home cage at 45 days of age, in the morning. The object was a 14 cm multicoloured cylinder (∅: 1.5 cm) covered with strips of grey, green, white, yellow and blue tape. A similar novel object was previously used by Schweitzer and Arnould [36]. The object was introduced into the cage gently by hand, clipped to the front of the cage and withdrawn after 10 min. The behaviour of each quail was recorded using digital camera placed in front of the cage so that the experimenter was out of the room during the test. The latency to peck at the novel object and the total number of pecks were measured.

### Physiological Measures

#### A. Body weight

Animals were weighed on the early afternoon at different moments of the experiment (Table 1): the day before the beginning of the URNS period (day 15), each week of the URNS period (day 27 and 38) and twice after the end of the URNS period (day 49 and 59). Quail were always fed with the normocaloric diet on these days. Body weight gain between day 17 and 27 as well as between day 28 and 38 were used to calculate feed conversion ratios which represent the amount of feed eaten related to body weight gain over a defined period.

#### B. Laying

The date of the first laying was noted for each quail, and the number of quail laying were noted daily for each group and each line.

### Statistical Analysis

All results were reported as means ± se. Kolmogorov-Smirnov tests determined whether data were normally distributed or not.

Behaviours related to reactivity to human, novel object and novel environment as well as food preferences assessed by the choice tests were analysed using non-parametric statistics. URNS effect, genetic effect, and URNS effect within each line were tested with Mann-Whitney tests. The relative preference for the hypercaloric diet, measured as a proportion of the total intake during preference tests, was compared to random (50%) using a Wilcoxon test to determine whether a preference existed.

Data related to feed intake (on the short term and daily), growth, and behavioural data that were normally distributed (reactivity to novel food and restraint in a crush cage) were analysed with ANOVAs. ANOVA with repeated-measures was used to test the period effect and its interaction with the genetic line and URNS effects on feed intakes. Because the period effect was highly significant for most of the intakes and because interactions between the URNS and the period effects were also significant, results for period 1 and 2 were analysed and presented separately. A two-way ANOVA was then used to test the effects of the genetic line, the URNS and their interaction. When the p-value of the interaction between the effects was below 0.1, one-way ANOVA (group effect) and Fisher’s PLSD tests were performed to test the effect of URNS within each line. Comparisons between hypo and hypercaloric diet mean intakes during each period were tested with t-paired tests within each group.

The number of laying quail and the number of quail pecking at the novel object were analysed using the Chi_2_ test.

In all statistical tests, P<0.05 was considered as significant.

## Results

### Emotional Reactivity

#### A. Reactivity to human

**Figure 1:**
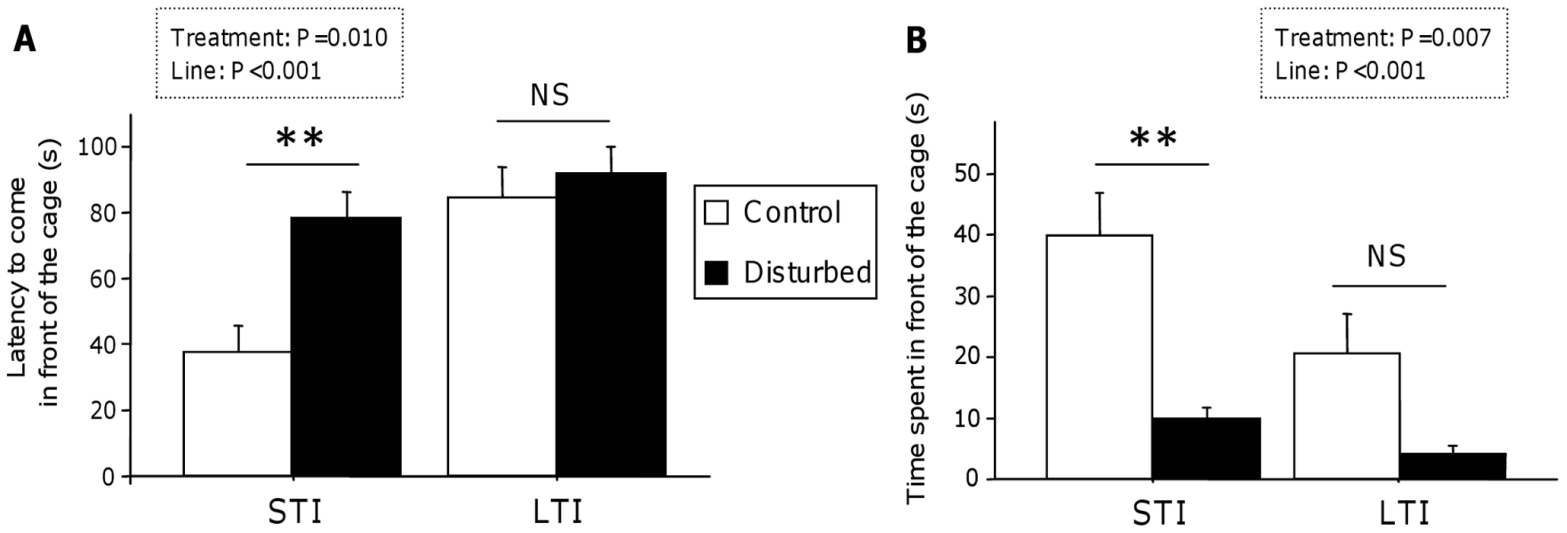
Quail’s reactivity to human. (A) LTI quail showed a higher latency to come in front of the cage (close to the human) compared to STI quail. The exposure to URNS led to an increase in the latency to approach the human in STI disturbed quail compared to STI control quail, whereas it had no effect on LTI quail. (B) LTI quail stayed less in front of the cage compared to STI line. The exposure to URNS decreased the time spent in front of the cage in STI quail whereas it had no effect in LTI quail. Data represent mean+se. Mann-Whitney U tests: **, P<0.01; NS, P≥0.1.

The latency to come in front of the cage (*i.e.* close to the human being) and the time spent in front of it depended on the genetic line: LTI quail came later and stayed less in front of the cage than STI quail (Mann-Whitney U tests: U = 1360.5, P<0.001 and U = 1277, P<0.001 respectively; Fig. 1a and 1b).

URNS induced changes in the behaviour of the quail with the disturbed quail coming later in front of the cage (Mann-Whitney U test: U = 1513, P = 0.011; Fig. 1a) and spending less time in front of it than the control quail (Mann-Whitney U test: U = 1479, P = 0.007; Fig. 1b).

This URNS effect was observed in the STI line (Mann-Whitney U tests: U = 257, P<0.001 for the latency to come in front of the cage; U = 276, P = 0.002 for the time spent in front of the cage; Fig. 1a and 1b) but not in the LTI line where control and disturbed quail expressed similar reactivity to human (Mann-Whitney U tests: U = 481, P = 0.632 for the latency to come in front of the cage; U = 460, P = 0.422 for the time spent there; Fig. 1a and 1b).

#### B. Reactivity to a novel object

**Figure 2:**
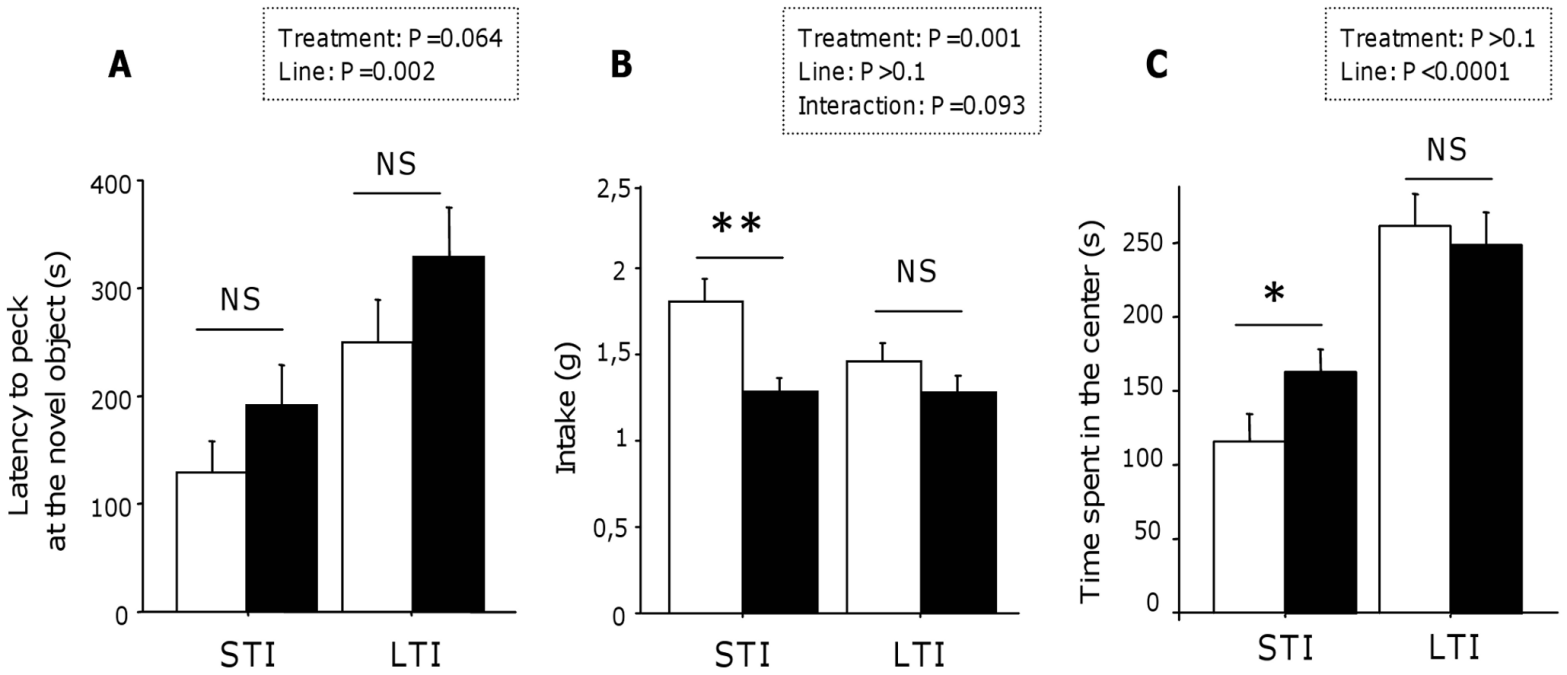
Quail’s reactivity to a novelty. (A) Reactivity to a novel object: The latency to peck at the novel object was higher for the LTI quail compared to the STI quail, and it tended to increase with the exposure to URNS when considering all the quail. Data represent mean+se. Mann-Whitney U tests: NS, P≥0.1. (B) Reactivity to a novel food: The genetic line did not influence the amount of novel food consumed by the quail, but the exposure to URNS decreased the amount of novel food consumed in STI quail. Data represent mean+se. Two-way ANOVA: **, P<0.01; NS, P≥0.1. (C) Reactivity to a novel environment: The STI quail spent less time in the center of the open field compared to the LTI quail. The exposure to URNS induced an increase of the time spent in the center of the open field in STI quail, but had no effect on LTI quail. Data represent mean+se. Mann-Whitney U tests or Fisher’s test: *, P<0.05; NS, P≥0.1. White bars: control group. Black bars: disturbed group.

The number of LTI quail pecking at the novel object (n = 47) was lower than the number of STI quail pecking at it (n = 58) (Chi2 = 6.454, P = 0.011). The genetic line also influenced the latency to peck at the novel object with the LTI quail pecking at the novel object later than the STI quail (Mann-Whitney U test: U = 1368, P = 0.002; Fig. 2a).

The URNS tended to decrease the number of quail pecking at the novel object (48 disturbed quail vs. 57 control quail; Chi2 = 2.835, P = 0.092) and tended to increase the latency to peck at the novel object (Mann-Whitney U test: U = 1633, P = 0.064; Fig. 2a).

#### C. Reactivity to a novel food

It tended to have an interaction of the genetic line and the URNS for the amount of novel food consumed (two-way ANOVA: F_(1,125)_ = 2.860, P = 0.093; Fig. 2b). The genetic line did not influence the amount of novel food consumed by the quail (two-way ANOVA: F_(1,125)_ = 2.620, P = 0.108; Fig. 2b). Disturbed quail consumed less the novel food than the control quail (two-way ANOVA: F_(1,125)_ = 11.118, P = 0.001; Fig. 2b). Control quail from the STI line were the one who consumed the highest amount of novel food when compared to each other groups (Fisher’s PLSD tests: P<0.05; Fig. 2b); the STI control, LTI control and LTI disturbed groups showed similar responses when compared to each other (Fisher’s PLSD tests: P>0.1; Fig. 2b).

#### D. Reactivity to a novel environment

LTI quail spent more time in the centre of the open field arena, *i.e.* their initial position in the arena, compared to STI quail (Mann-Whitney U test: U = 893.5, P<0.0001; Fig. 2c). Disturbed and control quail showed similar time spent in the centre of the open field arena (Mann-Whitney U test: U = 1844, P = 0.492; Fig. 2c). This is true in the LTI line (Mann-Whitney U test: U = 469, P = 0.557; Fig. 2c) whereas in the STI line, disturbed quail stayed longer in the centre of the open field arena compared to control quail (Mann-Whitney U test: U = 318.5, P = 0.023; Fig. 2c).

#### E. Restraint in a crush cage

There was no interaction between the genetic line and the URNS for the number of body movements in this test (two-way ANOVA: F_(1,125)_ = 1.365, P = 0.245).

**Figure 3:**
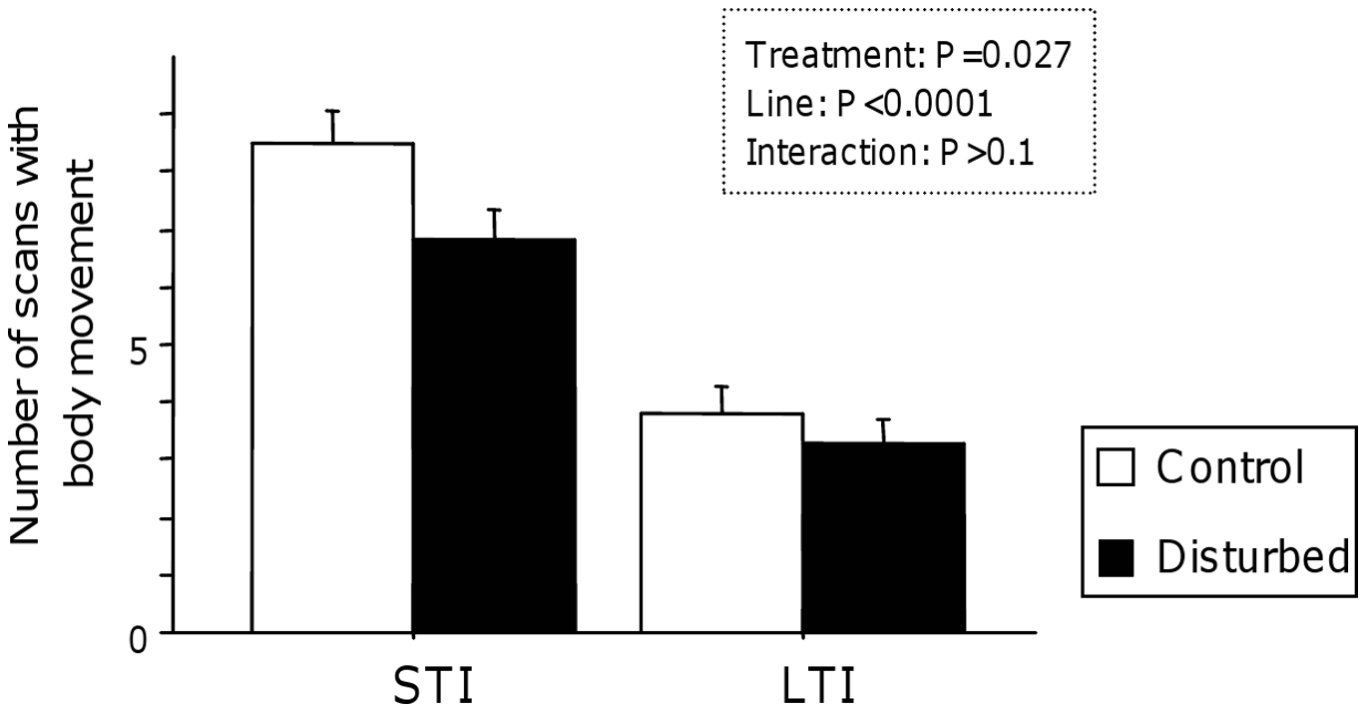
Quail’s response to a restraint in a crush cage. The number of scans with body movement was influenced by the genetic line (LTI quail were more inactive than STI quail) and by the exposure to URNS with disturbed quail more inactive than control quail. Data represent mean + se.

LTI quail showed less body movements during the restraint period compared to STI quail (two-way ANOVA: F_(1,125)_ = 68.143, P<0.0001; Fig. 3). In this test, URNS decreased the number of body movements (two-way ANOVA: F_(1,125)_ = 5.001, P = 0.027; Fig. 3).

### Feeding Behaviour

#### A. Feed preferences

Both lines highly preferred the hypercaloric diet compared to the hypocaloric diet over the 3 choice tests, *i.e.* the percentage of intake of the hypercaloric diet over the total intake during each choice test was higher than 50% (Table 2; Wilcoxon tests, z<−4.5, P<0.0001).

**Table 2:** Preferences for the hypercaloric diet (%, proportion of the total intake) measured over 3 two-way choice tests.

	STI line		LTI line		*P* *	
	Control	Disturbed	Control	Disturbed	Line	URNS
	m** ± se	m** ± se	m** ± se	m** ± se		
During the exposure to URNS						
End of the 1^st^ period (d27)						
	80.4±5.0	69.2±6.1	89.6±2.8	84.8±4.1	0.009	NS
End of the 2^nd^ period (d38)						
	84.1±4.1	90.7±3.4	81.4±5.2	78.7±6.2	NS	NS
After the exposure to URNS						
	71.0±4.9	65.2±7.0	79.7±5.2	78.7±5.6	0.041	NS

* Probability resulting from Mann-Withney U tests.

** Comparisons with random (50%) was significant for each mean (Wilcoxon paired test; P<0.0001).

LTI quail showed a higher preference for the hypercaloric diet compared to STI quail at the end of the first period (LTI quail, 87.2±2.5% *vs* STI quail, 74.9±4.0%; Mann-Whitney U test: U = 1474, P = 0.009) and ten days after the end of the URNS (LTI quail, 79.2±3.8% *vs* STI quail, 68.1±4.2%; Mann-Whitney U test: U = 1591, P = 0.041). The relative preference for the hypercaloric diet was however not affected by the genetic line during the choice test performed after the second period (LTI quail, 81.7±3.3% *vs* STI quail, 87.5±2.5%; Mann-Whitney U test, U = 1795, P = 0.437).

Disturbed and control quail showed similar preferences for the hypercaloric diet over the 3 choice tests (Table 2; Mann-Whitney U tests, U>1729, P>0.1).

#### B. Daily and total intake

There were no interactions between the genetic line and the URNS for all the parameters that concern daily and total intakes.

Over the first period, mean daily intakes of hypo and hypercaloric diets were neither affected by the genetic line nor by the URNS (Table 3). LTI quail showed a greater daily intake of the hypercaloric diet compared to the hypocaloric diet regardless of the URNS (Table 3; t paired test: t–paired value = 2.155, P = 0.039 for control LTI quail; t paired test: t–paired value = 3.310, P = 0.002 for disturbed LTI quail). The mean daily energy intake provided by the hypercaloric diet was higher than the one provided by the hypocaloric diet whatever the group (Table 3; t paired test for each group, t-paired values>8.386, P<0.001).

**Table 3:** Feed intake in control and disturbed quail.

	STI line		LTI line		*P* *	
	Control	Disturbed	Control	Disturbed	Line	URNS
	m ± se	m ± se	m ± se	m ± se		
Mean daily intake						
First period						
Hypo (g)	16.43±1.97	16.05±1.64	15.97±1.36x**	16.19±1.55x	NS	NS
Hyper (g)	16.73±1.91	16.15±1.66	16.56±1.41y	16.79±1.69y	NS	NS
Hypo (kJ)	192.3±23.0x	187.8±19.2x	186.9±15.9x	189.5±18.1x	NS	NS
Hyper (kJ)	223.8±25.5y	216.0±22.2y	221.5±18.9y	224.6±22.6y	NS	NS
Second period						
Hypo (g)	18.87±1.98	18.18±1.73	17.56±1.26x	16.91±1.31x	<0.001	0.023
Hyper (g)	19.08±1.44	17.97±1.34	17.89±1.22y	17.55±1.64y	0.003	0.005
Hypo (kJ)	220.8±23.2x	212.8±20.2x	205.5±14.7x	197.9±15.3x	<0.001	0.023
Hyper (kJ)	255.2±19.3y	240.4±17.9y	239.3±16.3y	234.7±21.9y	0.003	0.005
Total intake (g)						
First period	132.1±2.9	128.2±2.3	130.1±1.7	131.6±2.9	NS	NS
Second period	151.8±2.3	143.5±2.0	142.1±1.7	137.9±1.9	<0.001	0.002
Periods + interval	342.0±5.4	327.1±4.3	325.7±3.4	321.5±4.3	0.014	0.029

* Probability resulting from two-way ANOVA. The interactions between the effects of the genetic lines and the exposure to URNS are not mentioned since they were not significant (P>0.10).

** x and y indicate significant differences between hypo- and hyper-caloric diet intakes within periods and within groups (t paired test; P<0.05).

“Hypo” means the hypocaloric diet, and “Hyper” means the hypercaloric diet.

Over the second period, mean daily intakes of the hypo and hypercaloric diets were lower for the LTI quail compared to the STI quail and reduced by the URNS (Table 3). Mean daily intake of the hypercaloric diet was higher than that of the hypocaloric diet in disturbed LTI quail only (t paired test: t–paired value = 2.740, P = 0.010). As in the first period, mean daily energy intake provided by the hypercaloric diet was however higher than the one provided by the hypocaloric diet whatever the group (t paired test for each group, t-paired values>7.252, P<0.001).

Total intake over the first period was neither affected by the genetic line nor the URNS (Table 3). Total intake over the second period was however reduced in LTI quail compared to STI quail and also reduced by the URNS (Table 3). Total intake over the two periods added with the interval period was similarly affected by the genetic line and the URNS (Table 3).

#### C. Short-term intake

**Figure 4:**
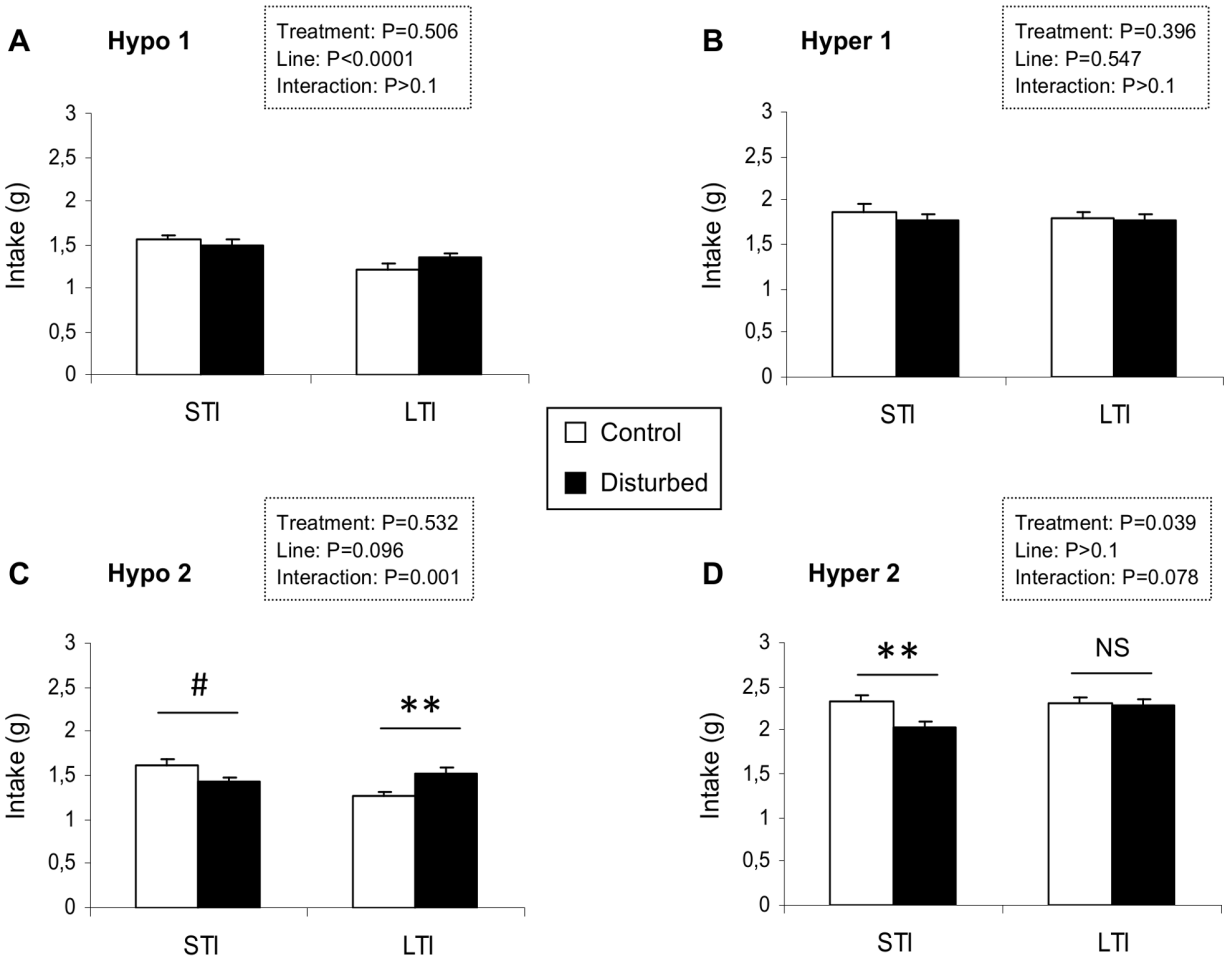
Quail’s motivation for the hypo and hypercaloric diets, over two periods. (A) Short-term intake (30 minutes) of the hypocaloric diet over the first period: the LTI quail showed a smaller motivation for the hypocaloric diet compared to the STI quail. (B) Short-term intake (30 minutes) of the hypercaloric diet over the first period: nor the genetic line neither the exposure to URNS influenced the motivation for the hypercaloric diet. (C) Short-term intake (30 minutes) of the hypocaloric diet over the second period: the exposure to URNS increased the motivation for the hypocaloric diet in LTI quail. (D) Short-term intake (30 minutes) of the hypercaloric diet over the second period: the exposure to URNS decreased the motivation for the hypercaloric diet in STI quail. Data represent mean+se. Fisher’s tests: **, P<0.01; NS, P≥0.1.

Over the first period, there was no interaction between the genetic line and the URNS concerning the intakes of both the hypocaloric and hypercaloric diet (Fig. 4a and 4b). The short-term intake of the hypocaloric diet was lower for the LTI quail compared to the STI quail (two-way ANOVA: F_(1,123)_ = 16.714, P<0.0001; Figure 4a) but it was not affected by the URNS (two-way ANOVA: F_(1,123)_ = 0.445, P = 0.506; Figure 4a). Concerning the short-term intake of the hypercaloric diet, it was neither modified by the genetic line (two-way ANOVA: F_(1,123)_ = 0.364, P = 0.547; Figure 4b) nor by the URNS (two-way ANOVA: F_(1,123)_ = 0.726, P = 0.396; Figure 4b).

Over the second period, there was an interaction between the genetic lines and the URNS on the short-term intake of the hypocaloric and a tendency for the hypercaloric diet (two-way ANOVA: Hypocaloric diet: F_(1,123)_ = 10.887, P = 0.001; Hypercaloric diet: F_(1,123)_ = 3.158, P = 0.078; Fig. 4c and 4d). The short-term intake of the hypocaloric diet was increased by the URNS for the LTI quail but unaltered for the STI quail (Fisher’s PLSD tests: LTI: P = 0.006 and STI: P = 0.062; Figure 4c). On the contrary, the short-term intake of the hypercaloric diet was decreased by the URNS for the STI quail (Fisher’s PLSD tests: P = 0.008; Figure 4d) but remained unchanged for the LTI quail (Fisher’s PLSD tests: P = 0.829; Figure 4d).

### Physiological Measures

#### A. Body weight

Quail from the STI line were lighter than quail from the LTI line at day 15, before the exposure to URNS (60.3±1.0 *vs.* 66.6±0.8 g, two-way ANOVA: F_(1,126)_ = 22.877, P<0.001) and at day 27, after the first period, (124.9±2.0 *vs.* 131.0±1.3 g, two-way ANOVA: F_(1,125)_ = 7.168, P = 0.008; Table 4). At day 37, there was no effect of the genetic line on the body weight of the quail (169.0±1.1 g, two-way ANOVA: F_(1,125)_ = 0.550, P = 0.460). At days 49 and 65, the effect of the genetic line was reversed and the LTI quail were lighter than the STI quail (186.3±1.9 *vs.* 193.7±2.0 g at day 49, two-way ANOVA: F_(1,125)_ = 7.046, P = 0.009; 211.5±2.8 *vs.* 229.5±2.4 g at day 65, two-way ANOVA: F_(1,125)_ = 23.686, P<0.001).

**Table 4:** Body weight (g) over the study.

	STI line		LTI line		*P* *	
	Control	Disturbed	Control	Disturbed	Line	URNS
	m ± se	m ± se	m ± se	m ± se		
Body weight (g)						
Before the exposure to URNS (d15)	61.1±1.6	59.5±1.4	67.4±1.2	65.8±1.20	<0.001	NS
During the exposure to URNS						
End of the 1^st^ period (d27)	127.8±2.6	121.8±3.0	132.3±1.8	129.7±1.90	0.008	0.068
End of the 2^nd^ period (d38)	173.6±2.4	166.0±2.5	169.7±2.1	166.7±2.10	NS	0.019
After the exposure to URNS						
At day 49	195.6±3.0	191.7±2.6	186.5±2.7	186.2±2.7	0.009	NS
At day 65	229.4±3.1	229.6±3.8	215.3±3.8	207.6±4.3	<0.001	NS

* Probability resulting from two-way ANOVA. The interactions between the effects of the genetic lines and the exposure to URNS are not mentioned since they were not significant (P>0.10).

At day 15, before any treatment, there was no difference between the disturbed and control groups (two-way ANOVA: F_(1,126)_ = 1.437, P = 0.233; Table 4). After one period of disturbances (day 27), URNS tended to decrease the body weight of the disturbed quail compared to the control group (two-way ANOVA: F_(1,125)_ = 3.391, P = 0.068). After two periods of disturbances (day 37), URNS decreased the body weight of the disturbed quail compared to the control group (two-way ANOVA: F_(1,125)_ = 0.5619, P = 0.019). Then, after one and two weeks without any disturbance, the disturbed and control quail showed similar body weight (two-way ANOVA: F_(1,125)_ = 0.573, P = 0.450 at day 49; F_(1,125)_ = 1.010, P = 0.317 at day 65).

Feed conversion ratio which represents the amount of diet eaten related to body weight gain, was not affected by the genetic line (ANOVA: F_(1,117)_ = 1.690, *P* = 0.196) nor by the URNS (ANOVA: F_(1,117)_ = 1.098, *P* = 0.297) over the first period (m± se = 2.57±0.18 g of diet/g body weight gain). LTI quail had a higher feed conversion ratio compared to STI quail, over the second period (5.17±0.62 *vs* 4.62±0.72 g/g; ANOVA: F_(1,118)_ = 19.310, *P*<0.0001) but there was no effect of the URNS (ANOVA: F_(1,118)_ = 0.012, *P* = 0.914) or interaction between both (ANOVA: F_(1,118)_ = 0.007, *P* = 0.936).

#### B. Laying

**Figure 5:**
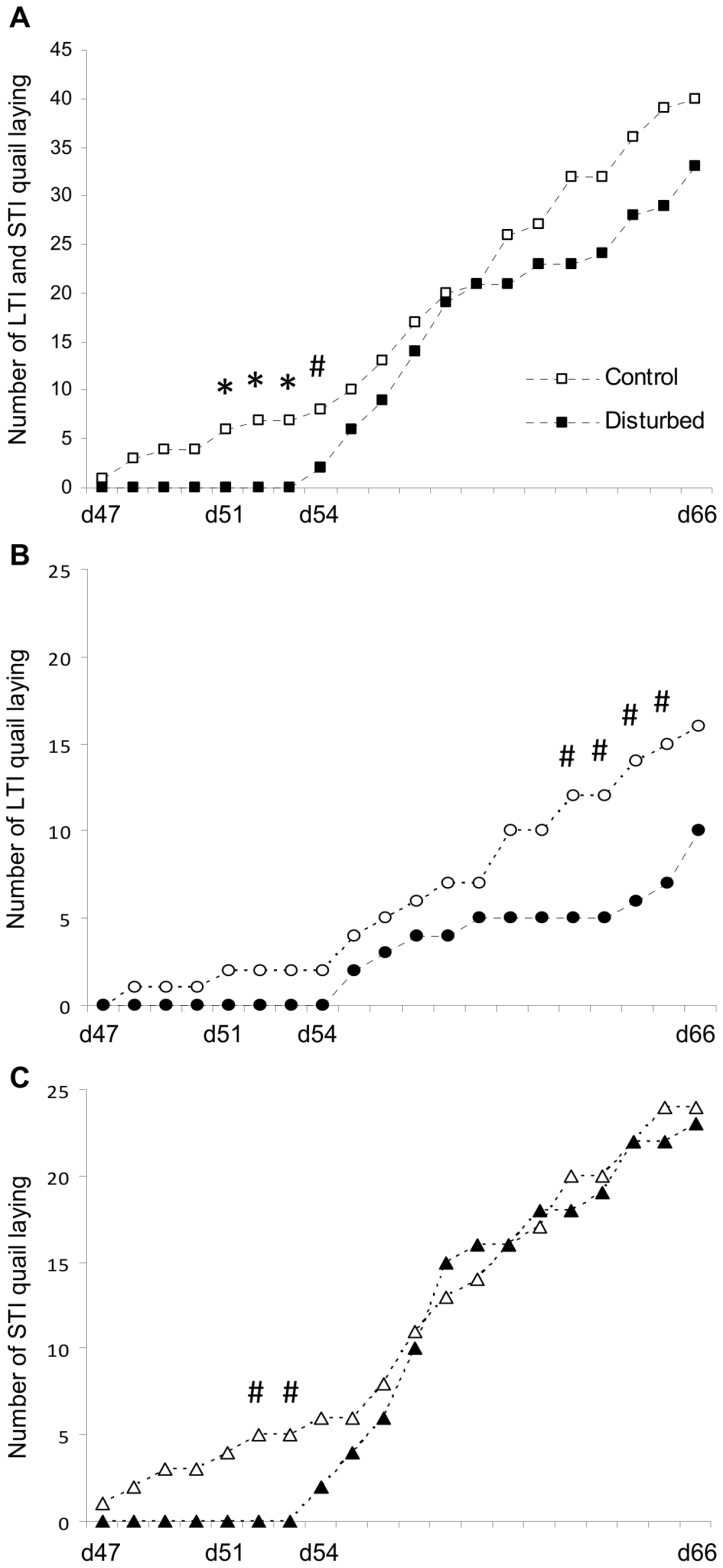
Consequences of URNS on laying. (A) In LTI and STI quail, the exposure to URNS altered laying as disturbed quail started laying later and were fewer to lay from day 51 to 54 compared to control quail. (B) In LTI quail, URNS decreased the number of quail laying although this only appeared on the last days from a statistical point of view. (C) In STI quail, URNS had an impact on laying at the beginning of the laying period. Chi-square tests: *, P<0.05; #, 0.05≤P<0.1.

The number of LTI quail laying was lower than that of the STI quail from day 47 to the end of the experiment; it was first a tendency at day 54 (Chi_2_ = 3.905, P = 0.096) and then it was statistically true from day 57 to the end (Chi-square tests: Chi_2_ value varying from 5.151 to 18.071 depending on the day, P<0.05). Concerning LTI and STI quail, the number of disturbed quail laying was lower than that of the control group from day 47 to the end of the experiment but it was statistically true only from day 51 to day 54 (Chi-square tests: Chi_2_ value varying from 3.905 to 7.405 depending on the day, P<0.05 for each day except at day 54, P = 0.099; Fig. 5a). When considering each line independently, the URNS effect was weaker (Fig. 5b for the LTI line and Fig. 5c for the STI line).

## Discussion

Our results showed that the URNS procedure induced changes on emotional reactivity, feeding behavior, growth and reproduction of quail. As expected, our results also showed a great influence of their inherent emotionality (genetic factors) on these parameters. This could account for their different sensitivity to URNS, thus leading to different experience of chronic stress.

### Different Internal State between Lines

All the behavioural tests performed in our study to assess the emotionality of the quail confirmed that differences do exist between the two genetic lines. The effect of the genetic line on animal’s emotionality is of great importance as it occurred whatever the environment in which the animals were reared, *i.e.* with or without the URNS procedure.

LTI quail avoided humans much more than STI quail as they approached later and stayed less in front of the cage (*i.e.* close to the experimenter). When restrained in a crush cage, LTI quail were less active than STI quail. LTI quail were also more reactive when faced to novelty, whatever the nature of the novelty (object, food or environment). Compared to STI quail, LTI quail showed a higher latency to peck at the novel object and stayed more in the centre of the open field arena, which was their initial location. Similar studies were performed on rats divergently selected for a high or a low emotionality. Rats selected for a high emotionality explored less a novel environment and a novel object, and waited longer before eating a novel food compared to the rats selected for a low emotionality [37]. Rodents with a low emotionality commonly coped with novel stimulations in an active manner, while those with a high emotionality did it in an inactive or passive manner [38].

### Different Feeding Behaviour between Lines

As mentioned above, many studies have shown differences on the emotionality of LTI and STI quail tested in various stressful situations. Less is known however about their feeding behaviour. In this study, STI and LTI quail expressed a great preference for the hypercaloric diet compared to the hypocaloric diet over the whole experiment. It thus seemed that quail were able to perform an associative learning between food and post-ingestive consequences; then, choosing the diet with the highest positive reward (i.e. the hypercaloric diet). This ability to develop food or flavour conditioned preferences is well-known in rodents [39], pigs [40], ruminants [41] and also in broiler chickens [34]. From the first period of the experiment, LTI quail showed a higher relative preference for the hypercaloric diet and a lower motivation to consume the hypocaloric diet compared to STI quail. Then, LTI quail may be more sensitive to their feeding environment compared to STI quail, which can help them learn the post-ingestive value of the different diets and adjust their feeding behaviour as a consequence. LTI quail also had a greater daily intake of the hypercaloric diet compared to the hypocaloric diet which means that they did not eat just to have constant energy intakes and suggests that they might search for a comfort food to compensate their high emotionality as rats do when they are faced with a stressful environment [14], [42].

LTI quail showed a higher body weight than STI quail at 15 days of age, and this difference persisted until 27 days of age which is in line with their usual growth (Leterrier, personal communication). At the end of the experiment, STI quail showed however a higher body weight compared to LTI quail. This is logical with the facts that STI quail showed a higher total daily intake and a higher feed conversion ratio in the second period, than LTI quail. STI broiler chickens also grew faster and had a higher body weight compared to LTI broilers [43]. The higher emotionality of LTI quail also had negative consequences on their laying performances. A parallel can be done with mammals as “calm” ewes showed a greater ovulation rate compared to “nervous” ewes [44]. Blache et al. [45] supposed that the animal’s emotionality can affect the energy partitioning between different functions, then decreasing the priority of reproduction.

Our study showed that LTI quail had a higher emotionality, layed later than STI quail and seemed to search for a comfort food. In a recent study, an “anxiogenic” trait was found in LTI quail [24]. Altogether, these results suggested that LTI quail might be experiencing a depressive like state even under control conditions but further investigations are needed to validate this hypothesis.

### Line Sensitivity to URNS

Our URNS procedure induced major effects on emotional reactivity. When faced with a novel object or restraint in a crush cage, the URNS procedure increased the emotional reactivity of the quail, whatever their genetic lines. The disturbed quail pecked at the novel object later than the control quail, and they were less active when restrained in a crush cage. In sheep, URNS also increased the animals’ emotional reactivity when they were submitted to a novel environment, a novel object and a human [3]. In other cases (reactivity to human, novel food, and novel environment), the URNS effect depended on the genetic line and appeared only in the STI line. In those cases, the disturbed STI quail showed a higher emotional reactivity compared to the control STI quail. It seemed that the emotional reactivity of the LTI quail in such fearful situations was so high that the URNS could not affect it more (*i.e.* ceiling effect). Then, LTI quail appeared to be more reactive than disturbed STI quail who were themselves more reactive than control STI quail. LTI control quail looked like disturbed birds even if they were not submitted to the URNS procedure. Previous studies using two-weeks chronic stress procedures also demonstrated that the inherent fearfulness of quail modulated the effect of URNS on their emotional reactivity (assessed with an open-field and/or an emergence tests), but contrary to our results, they showed that LTI quail were affected by the URNS while STI quail showed no differences with or without the URNS [27], [29]. It is then possible that the duration of the chronic stress procedure (2 weeks in [27], [29] vs. 4 weeks in our study) played a role on the intensity of the disturbance felt by the quail and thus modulate differently the effect of the URNS in each genetic line.

The URNS procedure altered the emotional reactivity of the quail, and this effect can be modulated by their genetic background as it influenced the inherent sensitivity of the birds to stressful events. It can thus be assumed that the URNS procedure succeeded to induce a state of chronic stress in disturbed quail.

### Chronic Stress and Feeding Behaviour

In animals and human, the effect of stress on feeding behaviour is characterized by a great individual variability [30] which can also be seen between the quail lines. In our study, quail expressed a great preference for the hypercaloric diet (measured in a short-term choice test with the hypocaloric diet) and, it was never decreased by the URNS procedure over the experiment. In the literature, results on anhedonia (decreased value of otherwise rewarding stimuli; [46]) and chronic mild stress procedure varied from a study to another. For instance, rodents submitted to a chronic mild and unpredictable stress procedure decreased their consumption and their preference for a highly palatable solution [47]. When the chronic mild stress procedure was paired with a massive decrease in body weight [48] or when female rats were submitted to such a chronic mild stress procedure [49], the results differed from the previous study and decreases in sucrose intake were not accompanied by decreases in sucrose preference. Such a variability in the anhedonic response to chronic mild stress procedure suggested that it might be useful to have other measures concerning the feeding behaviour of the birds. That was why we decided to look at the daily intake of the birds which involve metabolic regulations, and also their motivation to eat each diet (independently of any simultaneous comparison with the other diet as in the two-way choice test) assessed by their short-term intake. The absence of any effect of the URNS over the first period may mean that a certain amount of negative disturbances need to accumulate before affecting the animal. It may also be linked to the fact that this first period is more distant from the puberty. The susceptibility to stress is indeed increased during the peripubertal period because of large changes in the brain (see [50] for review). The URNS procedure had however some interesting effects on the daily intake and the motivation to eat (in interaction with the genetic line) over the second period. The disturbed STI quail showed a decrease in their daily intake of both the hypo and hypercaloric diets, and reduced their motivation for the hypercaloric diet compared to the STI control quail. If we consider the decrease in the motivation to eat a hypercaloric diet as an indirect measure of anhedonia and combined it with the decrease in the daily intake of both diets, it can be assumed that STI disturbed quail were in a devaluation process and that their emotional state might be close to a melancholic depressive-like state. Indeed, the American Psychiatric Association’s Diagnostic and Statistical Manual IV [51] describes two types of depression felt by the individual: a melancholic depression generally induces a decrease in daily intake and/or anhedonia while an atypical depression is generally responsible for an increase in daily intake. It was furthermore hypothesized that individuals who expressed atypical depression could try to feel better through comfort food because of its negative feedback on the activity of the hypothalamo-pituitary-adrenal axis [42]. Disturbed LTI quail showed a decrease in their daily intake. Their motivation for the hypocaloric diet was however surprisingly increased, compared to that of the control LTI quail, while the motivation to eat the hypercaloric diet was still high. The disturbed LTI quail ate less on a daily basis but, they were motivated to eat on the short term, whatever the diet, which suggested a short-term compensatory behaviour towards stressful events. The decrease in the daily intake of the hypercaloric food was unexpected as chronically stressed rats were found to decrease their intake of a standard food while increasing their intake of a palatable food (called comfort food) when they have the choice [14], [52]. It is then possible that the sequential feeding used in our study is not perceived as a real choice situation by the chronically stressed animals, thus preventing them to compensate their stress by over consuming the hypercaloric food on a daily basis. Taking into account that STI disturbed quail might be in a devaluation process whereas LTI disturbed quail seemed to express a compensatory behaviour, this study suggests that the reward system might be affected by URNS, which open up new perspectives for future researches.

The anorexigenic effect of the chronic stress was associated with a decrease in the body weight of the disturbed quail. The growth performance of broiler chickens was also depressed by chronic stress, mimicked by repeated corticosterone exposures, but this effect was not due to a decrease in daily food intake; the authors indeed assumed that it was due to an increase in energy expenditure because of a high feed consumption and a low feed efficiency measured in stressed broilers [53]. Different behavioural responses to chronic stress thus lead to similar effect on body weight. The loss in body weight was however not a long lasting effect as it did not persist after the end of the URNS procedure (recovery period). Similar results (decrease in body weight during the URNS period and regain during the recovery period) were found in Awerman and Romero [8]; these authors suggested that body weight decreases because of a temporary muscle wasting. The URNS procedure delay and reduce laying in quail as previously shown in hens [9]. From a biological point of view, these negative effects of the URNS procedure on body weight and laying might be due to the cost of the adaptation process.

## Conclusion

Our study confirms that differences do exist in the emotional reactivity of LTI and STI quail but it shows, for the first time, that these two genetic lines also differ in their feeding behaviour. In fact, LTI quail seemed to search for a comfort food as they showed a higher preference for the hypercaloric diet and a lower motivation for the hypocaloric diet compared to STI quail.

Coming back to the main objective of this study, unpredictable and repeated negative stimuli increased the emotional reactivity of quail and induced large changes in quail’s feeding behaviour although these changes differed between lines. LTI disturbed quail seemed to express a short-term compensatory behaviour because of their high motivation to eat, whereas STI disturbed quail seemed to be in a devaluation process as shown by their anhedonia and their decrease of daily intakes. Thus, URNS induced a chronic stress state in quail, but they exhibited various ways to cope with these negative events depending on their genetic background.

Chronic stress altered animal welfare but also decreased the profitability of the poultry breeding system as it decreased animal’s body weight and delay the onset of laying. The identification of chronic stressors in poultry breeding systems is thus of importance.
